# Effect of probiotics on the incidence of acute kidney injury in patients with severe pneumonia

**DOI:** 10.3389/fmed.2025.1596037

**Published:** 2025-05-21

**Authors:** Qiang Zhang, Xuefeng Song

**Affiliations:** ^1^Department of Critical Care Medicine, Peking University Third Hospital, Beijing, China; ^2^Department of Gastrointestinal Oncology, Shanxi Province Cancer Hospital/Shanxi Hospital Affiliated to Cancer Hospital, Chinese Academy of Medical Sciences/Cancer Hospital Affiliated to Shanxi Medical University, Taiyuan, Shanxi, China

**Keywords:** severe pneumonia, probiotics, acute kidney injury, critical care, renal complications

## Abstract

**Objective:**

This study employed a retrospective analysis to investigate whether probiotics were associated with a lower incidence of Acute Kidney Injury (AKI) in patients with severe pneumonia.

**Methods:**

We retrospectively analyzed patients with severe pneumonia who required mechanical ventilation and had no prior history of kidney disease.

**Results:**

Based on the inclusion and exclusion criteria, a total of 267 cases were included, comprising 190 patients who did not develop AKI and 77 patients who developed AKI. Statistically significant differences (*p* < 0.05) were observed between the AKI and non-AKI groups in terms of gender, CRP, PCT, PLT, SOFA score, ALB, Cr, and BUN. The proportion of probiotics use was lower in the AKI group, with a statistically significant difference between the two groups (*p* < 0.05). Using the occurrence of AKI as the dependent variable, a multivariate logistic regression analysis was performed including gender, CRP, PCT, PLT, SOFA score, ALB, Cr, and BUN. The analysis demonstrated that probiotics were associated with a lower incidence of AKI during ICU hospitalization in patients with severe pneumonia (*p* = 0.031). The results of the ordered logistic regression further indicated that probiotics use reduced the incidence of AKI (Coefficient = −0.7748, *p* = 0.006). Subgroup analysis revealed that probiotics were associated with a lower incidence of stage 1 AKI (OR: 0.4023, 95% CI: 0.1895–0.8540, *p* = 0.018) but not stage 2 (OR: 0.683, 95% CI: 0.2083–2.242, *p* = 0.53) or stage 3 AKI (OR: 0.9007, 95% CI: 0.2851–2.845, *p* = 0.859). Among the 146 patients in the probiotic group and 121 patients in the non-probiotic group, 44 patients developed stage 1 AKI, 15 developed stage 2 AKI, and 18 developed stage 3 AKI. Creatinine was selected for matching to balance the confounder. After propensity score matching, a total of 224 cases were matched (112 in each group). The incidence of stage 1 AKI (*p* = 0.019) remained statistically significant, while no significant differences were observed in the incidence of stage 2 AKI (*p* = 0.757) or stage 3 AKI (*p* = 0.757) between the two groups.

**Conclusion:**

Probiotics were associated with a lower incidence of stage 1 AKI in patients with severe pneumonia. However, our research was a single-center retrospective study, and multi-center randomized controlled studies would be needed in the future for validation.

## 1 Introduction

Severe pneumonia is a life-threatening lower respiratory tract infection characterized by rapid clinical deterioration and high mortality. In addition to respiratory failure, severe pneumonia may result in multi-organ dysfunction, including acute kidney injury, which significantly worsens prognosis. During severe inflammation, the composition of the gut microbiota undergoes significant alterations, which can disrupt microbial balance. This dysbiosis is associated with exacerbated local and systemic inflammation, increased uremic toxin concentrations, elevated intestinal permeability, and the development of endotoxemia and immune deficiencies (1). Probiotics have been shown to enhance intestinal barrier function, reduce the host pathogen load, competitively inhibit pathogenic bacteria, regulate host inflammatory responses (2), and modulate the immune system (3). Some studies suggest that probiotics may reduce the risk of sepsis and lower respiratory tract infections (4). However, researchers conducted a randomized controlled trial and found that probiotic administration did not reduce the incidence of ventilator-associated pneumonia (VAP), duration of antibiotic use, or length of ICU stay in critically ill patients (1). They believe that although critically ill patients exhibit a reduction in commensal microbiota, overgrowth of potential pathogens, and consequently, a highly perturbed microbial community (5), probiotics may not improve clinically significant outcomes associated with dysbiosis in such circumstances. Similarly, recent research has demonstrated that, in mechanically ventilated patients with severe conditions, probiotics did not reduce the incidence of ventilator-associated pneumonia compared to a placebo (1). Despite numerous studies exploring the effects of probiotics on various health outcomes, results remain conflicting. These discrepancies may arise from several factors, including differences in patient populations, probiotic strains used, and variations in study designs.

Currently, the management of acute kidney injury (AKI) caused by sepsis is primarily limited to treating the underlying cause (6), and in severe cases, addressing its complications through kidney replacement therapy (KRT) (7). Therefore, there is an urgent need to explore alternative therapeutic approaches for AKI. In 2023, researchers conducted a multicenter randomized controlled trial to investigate whether probiotics could promote renal function recovery during AKI in sepsis. The results demonstrated that administering probiotics for seven consecutive days was safe but did not increase the likelihood of renal function recovery among sepsis patients (8).

As AKI is a syndrome that elicits a strong systemic inflammatory response (9), it has been demonstrated that attenuating this response can improve renal function and mitigate parenchymal damage (10). Animal experiments have revealed a bidirectional relationship between host AKI and gut microbiota. The gut microbiota can significantly influence the prognosis of AKI, while AKI can also induce alterations in the gut microbiota. This interaction is termed the “gut-kidney axis.” The bidirectional relationship between the gut and kidney underscores the critical interplay between these two organs, with significant clinical implications. This inter-organ communication suggests potential therapeutic avenues via microbiota modulation. Modulating the microbiota through probiotic administration to ameliorate gut dysbiosis during AKI appears to improve the prognosis of patients with these conditions (11). Current research primarily focuses on whether probiotics can improve the prognosis of patients with severe pneumonia and whether they can promote the recovery of AKI after its onset. Despite growing interest in the gut–kidney axis, there is limited evidence on whether probiotic administration can prevent AKI in patients with severe pneumonia who have no prior renal disease. This retrospective study investigates whether probiotics use is associated with a reduced incidence of AKI in this patient population.

### 1.1 Study population

A retrospective study was conducted on patients with severe pneumonia admitted to Peking University Third Hospital from February 2021 to September 2023 who required mechanical ventilation.

### 1.2 Inclusion and exclusion criteria

Mechanically ventilated patients meeting the diagnostic criteria for severe pneumonia (12, 13) were included. AKI is defined by the Kidney Disease: Improving Global Outcomes (KDIGO) organization as an increase in serum creatinine (sCr) levels by ≥0.3 mg/dL (≥26.5 μmoL/L) within 48 h, or an increase in sCr to ≥1.5 times baseline, or urine volume <0.5 mL/kg/h for 6 h (14). Use of probiotics was before the development of AKI. A total of 267 patients met the inclusion criteria and were included in the final analysis. Patients were divided into the probiotic group and the non-probiotic group based on whether they received probiotics during their ICU stay.

### 1.3 Exclusion criteria

Patients were excluded if they (1) died within 24 h of admission [(8) patients], (2) were under 18 years of age [(6) patients], (3) had pre-existing renal insufficiency [(41) patients], or (4) had active malignancies [(44) patients].

### 1.4 Ethics statement

This study was a retrospective study that complied with medical ethical standards and was approved by the Medical Ethics Committee of Peking University Third Hospital (Ethics approval number: M20250221). Given the retrospective design, the requirement for informed consent was waived by the ethics committee.

### 1.5 Observation indicators

Clinical data within 24 h of enrollment were collected, including age, gender, comorbidities (diabetes, hypertension, hepatic insufficiency), C-reactive protein (CRP), procalcitonin (PCT), D-dimer, white blood cell count (WBC), Sequential Organ Failure Assessment (SOFA) score, Oxygenation Index (OI), Albumin (ALB), Creatinine (Cr), and Blood Urea Nitrogen (BUN). Data on use of probiotics and the development of AKI were collected throughout the patients’ ICU stay.

### 1.6 Statistical methods

Statistical analysis was performed using STATA 18 software (StataCorp, College Station, TX, USA). The Kolmogorov–Smirnov method was used to test the normality of measurement data. Normality was defined as a Kolmogorov–Smirnov test *p* > 0.05. Visual inspections (Q-Q plots and histograms) further validated this assumption. Normally distributed measurement data are presented as mean ± standard deviation (±s) and analyzed using the *t*-test. Non-normally distributed measurement data are presented as median (interquartile range) [M (QL, QU)] and analyzed using the Mann–Whitney U nonparametric test. Count data are presented as *n* (%) and analyzed using the *χ*^2^ test. We performed univariate analysis,statistically significant differences (*p* < 0.05) were found between the AKI and non-AKI groups in terms of gender, CRP, PCT, PLT, SOFA, ALB, Cr, and BUN. Then these variables were included the multivariate logistic regression model. Multivariate logistic regression was performed to identify the independent predictors of AKI and to evaluate the correlation between probiotic use and AKI incidence. This regression analysis allowed us to control for potential confounding variables and isolate the specific factors that significantly influence the occurrence of AKI. By including relevant covariates in the model, we aimed to obtain a more accurate understanding of the relationship between probiotics use and AKI incidence while accounting for other variables that may affect the outcome. Subgroup analysis was also performed based on AKI stages. Before propensity score matching (PSM), we compared baseline characteristics between the probiotic and non - probiotic groups. Only creatinine levels showed a statistically difference. Thus, we chose creatinine for matching to balance this confounder and enhance result accuracy. A 1:1 match was performed using the nearest neighbor matching method, with a caliper set at 0.05 times the standard deviation to ensure matching accuracy. This analysis was conducted to determine whether there were differences in the occurrence of AKI between the probiotic and non-probiotic groups. *p* < 0.05 was considered statistically significant.

## 2 Results

Based on the inclusion and exclusion criteria, a total of 267 cases were included, with 190 patients (71.16%) not developing AKI and 77 patients (28.84%) developing AKI. The AKI group had lower male proportion (12.99% vs. 26.31%, *p* = 0.018), higher CRP (120.1 mg/L vs. 74.26 mg/L, *p* = 0.0076), higher PCT (1.22 ng/mL vs. 0.51 ng/mL, *p* = 0.001), lower PLT (160 vs. 185, *p* = 0.0089), higher SOFA score (10 vs. 7, *p* = 0.001), lower ALB (29.8 g/L vs. 32.9 g/L, *p* = 0.008), higher Cr (111 vs. 67, *p* = 0.001), and higher BUN (11.21 vs. 7.845, *p* = 0.001). No significant differences were found in age, D - dimer, WBC, OI, or the rates of hypertension, diabetes, and liver dysfunction between the two groups (*p* > 0.05) (Table 1).

**Table 1:** Comparison of basic information between two groups.

Factors	Non -AKI (*n* = 190)	AKI (*n* = 77)	*χ*^2^/Z/t	*p*
Age (year)	68 (56, 75)	67 (55, 76)	0.002	0.965
Gender (M)	50 (26.31%)	10 (12.99%)	5.5875	0.018
CRP (mg/L)	74.26 (24.9, 157.5)	120.1 (49.6, 242)	7.136	0.0076
PCT (ng/mL)	0.51 (0.156, 3.26)	1.22 (0.417, 19.91)	15.181	0.001
D-2 (μg/L)	2, 750 (1,340, 6, 490)	2, 756 (1,236, 6,662)	0.008	0.973
WBC (×10^9^/L)	11.25 (8, 15.33)	10.94 (7.04, 17.06)	0.070	0.792
PLT (×10^9^/L)	185 (118, 245)	160 (84, 208)	6.850	0.0089
OI (mmHg)	176.13 (119.83, 236.57)	160 (102.86, 205.7)	2.554	0.11
SOFA	7 (6, 10)	10 (7, 12)	17.87	0.001
ALB (g/L)	32.9 (29.6, 35.8)	29.8 (26.5, 34.3)	11.13	0.008
Cr (μmol/L)	67 (53.88)	111 (87, 167)	58.186	0.001
BUN (mmol/L)	7.845 (5.315, 11.87)	11.21 (8.51, 16.49)	19.814	0.001
Hypertension	95 (50%)	45 (58.44%)	1.5656	0.211
Diabetes	30 (15.79%)	20 (%)	3.7343	0.053
Liver dysfunction	66 (34.74%)	33 (25.97%)	1.5487	0.213
Probiotics	97 (51.05%)	24 (31.17%)	8.74	0.003

Probiotics use was higher in the non - AKI group (51.05% vs. 31.17%, *p* = 0.003).

## 3 Multivariate logistic analysis

According to the statistical results, 121 patients received probiotics, among whom 24 patients (19.83%) developed AKI; 146 patients did not receive probiotics, among whom 53 patients (36.30%) developed AKI. The chi-square test results showed that the proportion of AKI occurrence was statistically significant between the two groups before adjustment (*χ*^2^ = 8.74, *p* = 0.003). Using the occurrence of AKI as the dependent variable, multivariate logistic analysis was performed incorporating indicators (gender, CRP, PCT, PLT, SOFA, ALB, Cr, and BUN) with statistical differences between the two groups.

As shown in Table 2, after adjusting for the aforementioned (gender, CRP, PCT, PLT, SOFA, ALB, Cr, and BUN) factors, gender, CRP, PCT, PLT, BUN and Sofa score showed no significant correlation with AKI occurrence (all *p* > 0.05).

**Table 2:** Results after multiple logistic regression.

Factors	Odds ratio	Std. err.	|z|	P > |z|	[95% conf. interval]
Gender	0.610	0.274	−1.10	0.272	0.253–1.471
CRP (mg/L)	1.000	0.001	0.23	0.816	0.997–1.004
PCT (ng/mL)	1.009	0.0081	1.22	0.223	0.994–1.026
PLT (×10^9^/L)	0.9992	0.0018	−0.41	0.683	0.9956–1.003
BUN (mmol/L)	0.9868	0.0234	−0.56	0.575	0.942–1.034
Cr (μmol/L)	1.0201	0.0046	4.37	0.000	1.011–1.029
ALB (g/L)	0.904	0.0327	−2.78	0.005	0.842–0.9707
Sofa	1.022	0.0612	0.36	0.716	0.9088–1.149
Probiotics	0.483	0.163	−2.16	0.031	0.2491–0.9357
Cons.	3.581	5.041	0.91	0.365	0.227–56.543

Cr levels were positively correlated with AKI occurrence (OR = 1.020, 95% CI = 1.011–1.029, *p* = 0.000). ALB levels were inversely correlated with AKI occurrence (OR = 0.904, 95% CI = 0.842–0.970, *p* = 0.005).

Probiotics use was associated with reduced AKI occurrence (OR = 0.483, 95% CI = 0.249–0.935, *p* = 0.031). Patients using probiotics had about half the odds of developing AKI compared to non - users.

## 4 Results of ordered logistic regression

According to the KDIGO criteria, AKI is classified into stages 1, 2, and 3. The results of the Ordered Logistic Regression (Table 3) indicate that Probiotic use was negatively associated with AKI stage (Coefficient = −0.775, Std. err. = 0.282, z = −2.74, *p* = 0.006). The 95% CI was −1.328 to −0.221, indicating that probiotics use was significantly related to lower AKI stages.

**Table 3:** Results of ordered logistic regression.

AKI-stage	Coefficient	Std. err.	z	P > z	[95% conf. interval]
Probiotics	−0.7748613	0.2824	−2.74	0.006	−1.328376 to −0.2213462
_cut1	0.5922356	0.1698625			
_cut2	1.671473	0.2080382			
_cut3	2.343554	0.2606744			

The Brant test was conducted to assess the proportional odds assumption of the ordered logistic regression model (X^2^ = 2.18, *p* = 0.3358), thus the ordered logistic regression model is appropriate for the data.

The log-likelihood of the intercept-only model is −235.709, while the log-likelihood of the full model is −231.770. The deviance (D) with 263 degrees of freedom is 463.539. The likelihood ratio test statistic (LR) with 1 degree of freedom is 7.879, with a *p*-value of 0.005, indicating the model is statistically significant compared to the intercept-only model.

## 5 Subgroup analysis by AKI stage

Using the occurrence of AKI stages as the dependent variable, multivariate logistic analysis was performed incorporating statistically significant indicators from the two groups mentioned above. The results (Table 4) showed that probiotics could reduce the incidence of stage 1 AKI (OR: 0.4023, 95% CI = 0.1895–0.8540, *p* = 0.018) but not stage 2 (OR: 0.683, 95% CI = 0.2083–2.242, *p* = 0.53) or stage 3 AKI (OR: 0.9007, 95% CI = 0.2851–2.845, *p* = 0.859).

**Table 4:** Subgroup analysis of AKI.

AKI-stage	Odds ratio	Std. err.	95%CI	Z	*p*
AKI-1stage	0.4023	0.1545	0.1895–0.8540	−2.37	0.018
AKI-2stage	0 0.683	0.414	0.2083–2.242	−0.63	0.53
AKI-3stage	0.9007	0.5285	0.2851–2.845	−0.18	0.859

## 6 Propensity score matching

**Figure 1:**
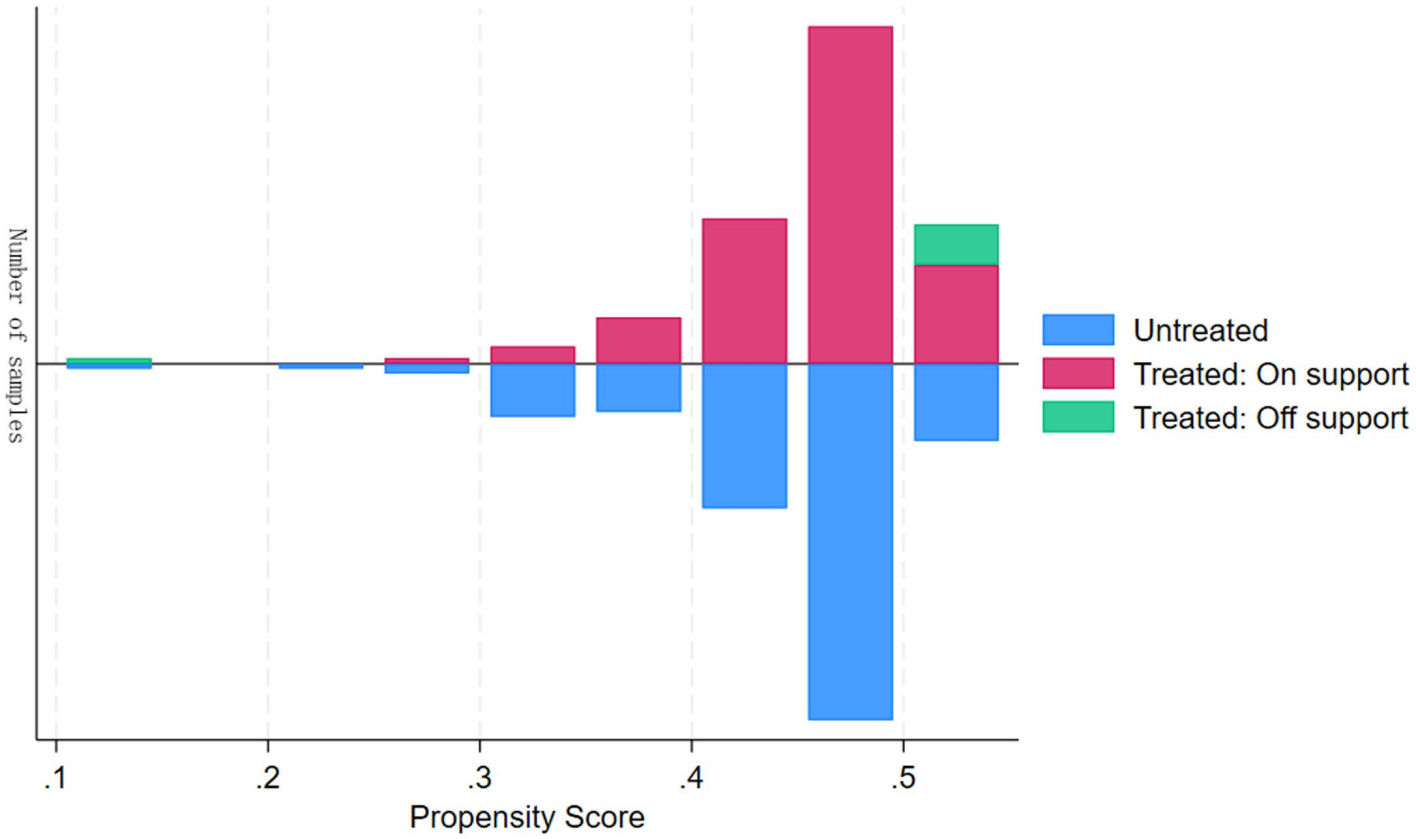
Graph of PSM.

Based on the highest AKI stage during the patient’s ICU stay, there were 44 patients with stage 1 AKI, 15 with stage 2 AKI, and 18 with stage 3 AKI. Before matching, there was a statistically significant difference in the incidence of stage 1 AKI between the two groups (*p* = 0.009); however, there were no statistically significant differences in the incidence of stage 2 AKI (*p* = 0.337) or stage 3 AKI (*p* = 0.570). We performed a univariate analysis of all potential covariates and found that only creatinine was significantly different between the probiotic and non-probiotic groups (*p* = 0.024). After propensity score matching with creatinine as a covariate, a total of 224 cases were matched (112 in the probiotic group and 112 in the non-probiotic group). Graph of PSM was shown in Figure 1. After matching, the two groups achieved good balance in baseline characteristics (Table 5). The occurrence of stage 1 AKI (*p* = 0.019) remained statistically significant. There were no statistically significant differences between the two groups for stage 2 AKI (*p* = 0.757) or stage 3 AKI (*p* = 0.757).

**Table 5:** Comparison of patient characteristics between probiotic group and non-probiotic group before and after PSM.

Factors	Before PSM			After PSM		
	Non-Probiotics (*n* = 146)	Probiotics (*n* = 121)	*p*	Non-Probiotics (*n* = 112)	Probiotics (*n* = 112)	*p*
Age (year)	67 (54, 75)	69 (56, 75)	0.521	67 (56, 75)	70 (56.5, 76)	0.3652
Gender (M)	35 (23.97%)	25 (20.66%)	0.519	28 (25.45%)	20 (18.18%)	0.193
SOFA	8 (6, 10)	7 (6, 10)	0.8227	7 (6, 10)	7 (6, 10)	0.4352
WBC (×10^9^/L)	12.06 (8.2, 16.48)	10.79 (7.4, 14.45)	0.0759	11.85 (8.38, 16.38)	10.86 (7.5, 14.89)	0.1922
CRP (mg/L)	92.95 (24.96, 182.6)	86.52 (30.75, 172.6)	0.756	92.95 (24.93, 155.89)	85.65 (30.23, 172.2)	0.9698
PCT (ng/mL)	0.735 (0.205, 5.06)	0.64 (0.17, 4.5)	0.7131	0.61 (0.167, 2.96)	0.65 (0.17, 5.1)	0.4658
D-2 (μg/L)	3,316 (1,470, 7,411)	2405 (1,130, 5,378)	0.1664	2,978.5 (1,315, 6,619.5)	2,380 (1,110, 5,378)	0.3822
PLT (×10^9^/L)	184.5 (111, 234)	172 (111, 231)	0.5489	191 (116, 244.5)	172.6 (102, 230)	0.2808
BUN (mmol/L)	8.52 (6.2, 13.87)	9.08 (5.73, 12.49)	0.6749	8.14 (5.59, 11.77)	9.38 (5.77, 12.68)	0.2537
Cr (μmol/L)	82 (59, 108)	71 (54.5, 106.5)	0.024	71.5 (55, 101)	73 (56.5, 107.05)	0.8069
ALB (g/L)	31.55 (28.06, 35.4)	32.85 (29.25, 35.65)	0.199	32.15 (28.13, 35.3)	33 (29.5, 35.8)	0.1892
OI (mmHg)	158.13 (104.29, 235)	179 (126, 222.5)	0.1396	168.99 (106.84, 237.08)	178.9 (124.58, 222.68)	0.5623
Hypertension	78 (53.42%)	62 (51.24%)	0.722	60 (54.545%)	58 (52.73%)	0.789
Diabetes	29 (19.86%)	21 (17.36%)	0.601	17 (15.45%)	19 (17.27%)	0.716
AKI-1	32 (%)	12 (9.92%)	0.009	25 (22.73%)	12 (10.91%)	0.019
AKI-2	10 (21.92%)	5 (4.13%)	0.337	6 (5.45%)	5 (4.545%)	0.757
AKI-3	11 (7.53%)	7 (5.785%)	0.570	5 (4.545%)	6 (5.45%)	0.757

To demonstrate balance after PSM, we calculated the standardized mean difference (SMD) for creatinine levels between the matched groups. The SMD was found to be −0.00286, this ensures that the matched groups are comparable, thereby strengthening the validity of our findings.

## 7 Discussion

Among mechanically ventilated patients with severe pneumonia, we found that probiotics can reduce the incidence of stage 1 AKI. Our findings add new evidence to the clinical application of probiotics. AKI presents significant challenges in pediatric care (15). Thus future similar perspective studies could be also made in children.

Common causes of AKI in patients with severe pneumonia include drug-induced nephrotoxicity (16), extra-renal tissue damage (17), and ischemia–reperfusion injury (IRI) (18). Studies have shown that oxidative stress (19), inflammation [unreferenced], mitochondrial damage (20), and immune dysfunction (21) are core mechanisms leading to the development and progression of AKI.

Recent studies have demonstrated that probiotics can mitigate the severity of gastrointestinal adverse reactions induced by antitumor treatment and restore disrupted gut microbiota (22). In a definitive efficacy trial, probiotics were employed as an adjunctive therapy for patients with depression (23). Uremic toxins derived from gut dysbiosis can exacerbate renal dysfunction and fibrosis. For instance, the gut-derived metabolite trimethylamine-N-oxide (TMAO) directly contributes to the progression of renal dysfunction and is associated with an increased risk of mortality (24). Certain probiotics prevent AKI by reducing inflammation and lowering oxidative stress levels. *Lactobacillus salivarius* BP121 exerts renoprotective effects primarily by modulating tight junction protein assembly through AMPK and TLR4 pathways and inhibiting the production of uremic toxins such as indoxyl sulfate (IS) and p-cresyl sulfate (PCS) (25). In mice with *Escherichia coli*-induced pyelonephritis, probiotic administration before and after sepsis significantly improved renal function, reduced inflammation, and attenuated renal fibrosis (26). Probiotics competitively inhibit pathogenic bacteria, maintain gut microbiota balance, and reduce the translocation of toxins and inflammatory mediators into the bloodstream. Additionally, they enhance gut epithelial barrier function, decrease endotoxin and bacterial translocation, and attenuate systemic inflammatory responses.

After AKI onset, probiotics have demonstrated efficacy in improving renal function, potentially by lowering creatinine and urea levels and significantly reducing pro-inflammatory cytokines like IL-1β, IL-6, TNF-*α*, and MCP-1 (27). They modulate immune cell activity, curb the release of pro-inflammatory cytokines (e.g., TNF-α, IL-6), and mitigate systemic inflammatory responses, while also boosting anti-inflammatory cytokines (e.g., IL-10) to alleviate inflammatory kidney damage. Probiotics enhance immune function and infection resistance by activating immune cells such as macrophages and T-cells. Gut dysbiosis can heighten uremic toxin production and foster AKI development (27). In a mouse renal ischemia–reperfusion injury (IRI) model (28), the probiotic *Bifidobacterium bifidum* (BGN4) increased gut microbiome evenness and curbed the proliferation of AKI-linked bacteria like Enterobacteriaceae and Bacteroidaceae. BGN4 intervention notably reduced tubular damage scores, neutrophil and macrophage infiltration, and renal IL-6 mRNA expression in mice (28). Additionally, BGN4 lowered plasma low-density lipoprotein, alanine aminotransferase, and aspartate aminotransferase levels, indicating its potential to alleviate renal IRI and AKI-related liver damage (29).

Additionally, some probiotics, such as ATCC 4356, can activate the nuclear factor erythroid 2-related factor 2 (Nrf2)/heme oxygenase-1 (HO1) axis, a crucial pathway for antioxidant stress, reduce the expression of malondialdehyde (MDA), and promote the expression of superoxide dismutase (SOD) and Nrf2. ATCC 4356 also exerts anti-inflammatory effects by regulating the PI3K/Akt signaling pathway (30).

Complement system is considered a key pathogenic mediator in the development of several renal diseases (31). Kidney is particularly susceptible to complement-mediated injury mainly due to its ultrafiltration function, low expression of complement regulatory factors, and local complement production (32). It can cause kidney injury and also cause AKI. Higher inflammation can lead to a hyperactive complement system, which can damage the kidney. Probiotics may influence the activation and regulation of complement components. This helps to reduce the release of factors that trigger complement activation.

Our study found that probiotics reduced the incidence of stage 1 acute kidney injury (AKI) but were ineffective for stage 2 and stage 3 AKI. This may be attributed to the fact that stage 1 AKI is milder, allowing probiotics to effectively intervene by modulating the gut microbiota and reducing inflammation. In contrast, stage 2 and stage 3 AKI are more severe and involve more complex pathological mechanisms, making single-intervention strategies such as probiotics insufficient. Probiotics primarily exert their effects by modulating the gut microbiota, reducing inflammation, and mitigating oxidative stress, which may be particularly effective in the early stages of AKI. In late-stage AKI, however, widespread systemic inflammation and multi-organ damage occur, and the effects of probiotics may be inadequate to reverse these complex pathological changes. Additionally, in stage 1 AKI, gut barrier function may remain relatively intact, enabling probiotics to exert their beneficial effects. In stage 2 and stage 3 AKI, severe damage to the gut barrier may hinder the ability of probiotics to function effectively. The immune response in stage 1 AKI is also milder, and probiotics may alleviate renal damage by modulating the immune system. In contrast, the immune response in late-stage AKI is more intense, and the regulatory effects of probiotics may be insufficient to control excessive immune activation. Moreover, the dosage and duration of probiotic administration may be adequate for stage 1 AKI but may require higher doses or extended treatment periods to address the severity of stage 2 and stage 3 AKI. Furthermore, patients with stage 2 and stage 3 AKI often present with additional complications or receive multiple concurrent treatments, which may interfere with the efficacy of probiotics.

Severe pneumonia and sepsis are closely related and interconnected. Pneumonia, especially severe pneumonia, is one of the most common causes of sepsis. The pathogenetic mechanisms of both diseases are similar in that they both involve the body’s immune response to infection, which can result in tissue damage and organ dysfunction. Additionally, sepsis patients are also at an increased risk of developing pneumonia, due to their weakened immune systems and the use of invasive medical devices. In clinical practice, a significant percentage of pneumonia cases are misdiagnosed as septic shock, and severe sepsis is often identified as a critical complication of pneumonia. Therefore, given the close relationship between severe pneumonia and sepsis, we shift our focus from severe pneumonia to sepsis to further explore the relevant research questions.

### 7.1 Limitations

Firstly, the sample size of this study is relatively small, particularly for stage 2 and stage 3 AKI cases. A larger sample size is required to enhance statistical power. Secondly, our data were derived from a retrospective study, and a randomized controlled trial (RCT) is recommended to further validate the effects of probiotics. As our study adopts an observational design, despite multivariate and propensity score matching (PSM) adjustments, there may still be residual confounding factors influencing the results. Moreover, the absence of microbiota profiling data, cytokines and claudin weakens the support for our mechanistic claims about the potential renal - protective effects of probiotics. Thirdly, the dosage, type, and duration of probiotics administration need to be further clarified. In clinical practice, there are still many issues that need to be addressed such as how to determine the optimal timing of probiotic intervention (whether it is better to administer probiotics early in the onset of AKI to prevent or mitigate kidney damage or at a specific stage of AKI progression), how to optimize probiotic dosage selection (tailoring dosage schemes according to patients’ individual characteristics, disease severity, and dynamic changes in microbiota), and how to stratify patients according to AKI stages to better assess the efficacy and safety of probiotics in different patient groups. Fourth, while these animal experimental results provide biological plausibility for the potential role of probiotics in AKI, extrapolating these findings to ICU patients with severe pneumonia carries certain risks.

## 8 Conclusion

Probiotics were associated with a lower incidence of stage 1 AKI in patients with severe pneumonia. However, our research was a single-center retrospective study, and multi-center randomized controlled studies would be needed in the future for validation. Defining optimal probiotic strains, dosages, and treatment windows would enhance the translational impact of the conclusions in future studies.

## Data Availability

The raw data supporting the conclusions of this article will be made available by the authors, without undue reservation.
